# Isolation and Characterization of a Novel Cold-Active, Halotolerant Endoxylanase from *Echinicola rosea* Sp. Nov. JL3085^T^

**DOI:** 10.3390/md18050245

**Published:** 2020-05-06

**Authors:** Jianlong He, Le Liu, Xiaoyan Liu, Kai Tang

**Affiliations:** 1 Department of Chemistry and Chemical Engineering, Huaiyin Normal University, Huaian 223300, China; jlhe@hytc.edu.cn (J.H.); lxy@hytc.edu.cn (X.L.); 2 State Key Laboratory of Marine Environmental Science, Fujian Key Laboratory of Marine Carbon Sequestration, College of Ocean and Earth Sciences, Xiamen University, Xiamen 361000, China; liulezz3@163.com

**Keywords:** xylanase, endoxylanase, cold-active, halotolerance, marine, *Echinicola rosea*

## Abstract

We cloned a xylanase gene (*xynT*) from marine bacterium *Echinicola rosea* sp. nov. JL3085^T^ and recombinantly expressed it in *Escherichia coli* BL21. This gene encoded a polypeptide with 379 amino acid residues and a molecular weight of ~43 kDa. Its amino acid sequence shared 45.3% similarity with an endoxylanase from *Cellvibrio mixtus* that belongs to glycoside hydrolases family 10 (GH10). The XynT showed maximum activity at 40 °C and pH 7.0, and a maximum velocity of 62 μmoL min^−1^ mg^−1^. The XynT retained its maximum activity by more than 69%, 51%, and 26% at 10 °C, 5 °C, and 0 °C, respectively. It also exhibited the highest activity of 135% in the presence of 4 M NaCl and retained 76% of its activity after 24 h incubation with 4 M NaCl. This novel xylanase, XynT, is a cold-active and halotolerant enzyme that may have promising applications in drug, food, feed, and bioremediation industries.

## 1. Introduction

Hemicellulose, a highly abundant storage polysaccharides found in plants, accounts for 15–35% of the dry weight of plant cells [1]. It is mainly composed of xylan-based hemicellulose, which contains β-d-xylopyranosyl residues attached by β-1,4-glycosidic bonds [2]. Based on different substitutions on the β-d-xylopyranosyl backbone, xylan can be categorized into homoxylan, arabinoxylan, glucuronoxylan, and arabinoglucuronoxylan [3].

Several xylanolytic enzymes, such as α-d-glucuronidase, endo-β-1,4-d-xylanase, α-l-arabinofuranosidase, β-d-xylosidase, and acetylesterase are needed for the complete hydrolysis of xylan [4]. Of these, the endo-β-1,4-d-xylanase enzymes (EC 3.2.1.8) hydrolyzes the β-1,4-glycosidic bonds in the xylose chain. The endoxylanases are classified based on their three-dimensional catalytic domain, as can be determined by their amino acid sequences [5]. In the Carbohydrate-Active Enzyme (CAZy) database, endoxylanases are classified into glycoside hydrolase (GH) families 5, 8, 10, 11, 16, 26, 30, 43, 51 62, 98, and 141 [6], and the predominant families are the GH10 and GH11. The endoxylanases in the GH10 family possess a (β/α)8-barrel fold in their molecular structures, and the endoxylanases in GH11 family have a β-jelly roll fold [7,8].

The metagenomic extraction of xylanases from variable sources, such as bacteria, yeast, fungi, and protozoa have been conducted, and metagenomic extraction using a culture-independent approach, and the environmental DNA of goat rumen contents have also been performed [9,10,11,12,13,14]. Xylanases have been utilized in many industrial and agricultural applications [15]. For instance, thermostable xylanases isolated from thermophilic microbes found in a hot spring have been utilized in the paper industry in various processes, including the kraft process and bio-bleaching of pulp [16,17]. Halotolerant xylanases have also been shown to have better efficiency when used in waste water treatment and paper industry [18,19]. Mesophilic xylanases, the most abundant type of xylanases, are used for clarifying fruit juices, for improving the consistency of beer, and for digesting animal feed stock [20,21]. Some textile and food-based industrial processes, as well as bioremediation, are carried out at low temperatures to stabilize product and avoid product denaturation [8]. Thus, some psychrophilic xylanases have been cloned from organisms found in streambed, Antarctic marine sponges, and seawater [9,10,22], and used in these low-temperature processes. The cold-active, halotolerant xylanase make an excellent additive for the detergent industry [23]. The enzymatic production of prebiotic oligosaccharides has gained more and more interest among researchers. Oligosaccharides can be utilized by the human gut microorganisms, such as *Lactobacillus* and *Bifidobacterium*, resulting the improvement of human health [24]. Functional oligosaccharides, including xylo-oligosaccharides (XOS), are difficult to digest in the human small intestine, due to the lack of corresponding digestive enzymes, but they are easily assimilated and metabolized by colon probiotic microorganisms, such as *Lactobacillus* and *Bifidobacterium*, so they are considered probiotics. Xylanase is an excellent tool to prepare XOS.

Marine xylanases exhibit some appealing characteristics, including cold adaption, hyperthermostability and halotolerancy, and the absence of cellulase activity, which differentiate them from terrestrial xylanases [22,25,26]. Therefore, marine xylanase can be operated in a wider scope of pH, temperature, and salt concentrations. Herein, we cloned a novel GH10 endoxylanase gene from a halotolerant marine bacterium *Echinicola rosea* sp. nov. JL3085^T^ [27], and recombinantly expressed it in *E. coli*. Characterization of the purified recombinant enzyme showed that it is a cold-active and halotolerant endoxylanase. This study is the first report on the identification of an endoxylanase isolated from the genome of a bacterium in the genus *Echinicola*, which contains an extremely large number of GH genes [28].

## 2. Results and Discussion

### 2.1. Production and Purification of Recombinant XynT

The *xynT* gene (WP_137405116.1), which encodes 379 amino acid residues of a putative endoxylanase, was cloned from the genomic DNA of *Echinicola rosea* sp. nov. JL3085^T^. This protein had a theoretical molecular weight of 43275.95 Da, and its isoelectric point was 4.93. After expression and purification of the recombinant XynT, the purified fractions were pooled and then subjected to enzymatic assays.

### 2.2. Biochemical Characterization of the Recombinant XynT

**Figure 1:**
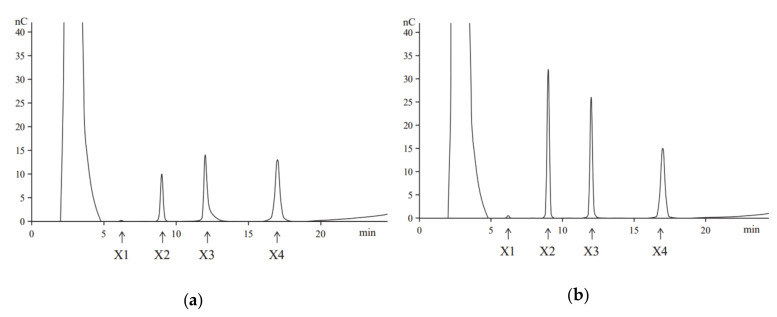
The high-performance anion-exchange chromatography (HPAEC) analysis of the hydrolysis of products of beechwood xylan performed at 40 °C for 2 h (**a**) or 8 h (**b**). Arrows indicate the positions of oligosaccharides: xylose (X1), xylobiose (X2), xylotriose (X3), and xylotetraose (X4).

Hydrolysis of beechwood xylan by the purified recombinant XynT revealed that the primary products were xylotetraose, xylotriose, and xylobiose, and the monosaccharide xylose (X1) was produced at a very low amount, even at a prolonged hydrolysis time (Figure 1). Similar products, but with different concentrations, were obtained when birchwood xylan was used in the hydrolysis (data not shown). These results confirm that the recombinant XynT is a typical endoxylanase, which has no β-xylosidase activity. XynT produce smaller oligosaccharides without producing xylose, and thus, is suitable for the production of XOS.

**Figure 2:**
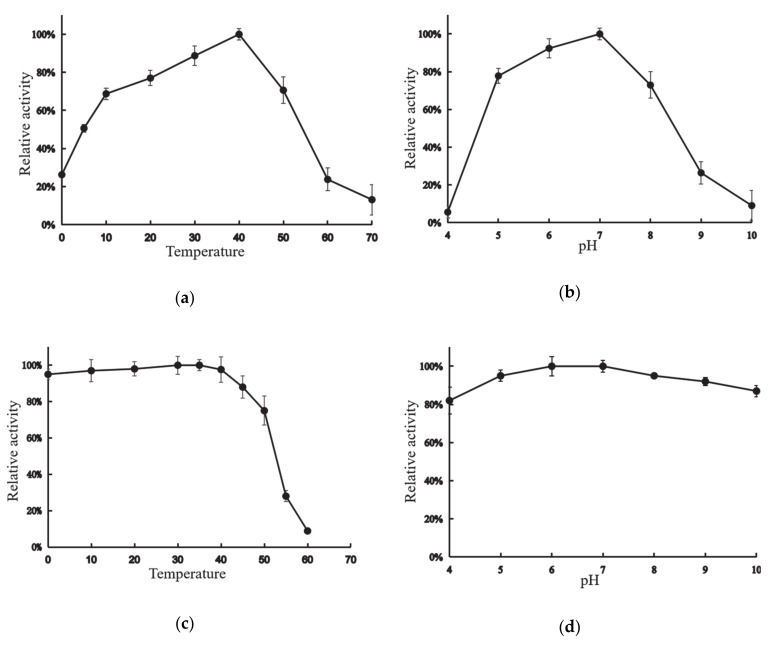
Activity profile of the xylanase gene (XynT) at various pH and temperatures. (**a**) XynT activity at pH 7 at various temperatures. (**b**) XynT activity at 40 °C at a different pH. (**c**) Effect of temperature on the stability of XynT. The residual activity of the enzyme was measured after incubation at 30–60 °C for 2 h, and the residual activity was then assayed under standard conditions. (**d**) Effect of pH on the stability of XynT. The residual activity of the enzyme was measured after incubation at pH 4–10 for 2 h.

XynT exhibited the maximum activity at 40 °C when beechwood xylan was used as the substrate (Figure 2a). The enzyme could retain its maximum activity by more than 69%, 51%, and 26% at 10 °C, 5 °C, and 0 °C, respectively. Figure 2b shows the effects of pH on the activity of XynT. XynT was found to be a neutral xylanase that showed optimal activity between pH 6–7, and was enzymatically active at pH 5–8, as it retained activity at above 70% at this pH range.

XynT was stable at 0–40 °C, but after incubation for 2 h at 50 °C, XynT lost its activity by approximately less than 25%, and almost completely lost all of its activity after incubation for 2 h at 60 °C (Figure 2c). Additionally, XynT was stable at pH 4–10; it could retain more than 82% of its maximum activity after incubation at 4 °C for 2 h at this pH range (Figure 2d).

**Figure 3:**
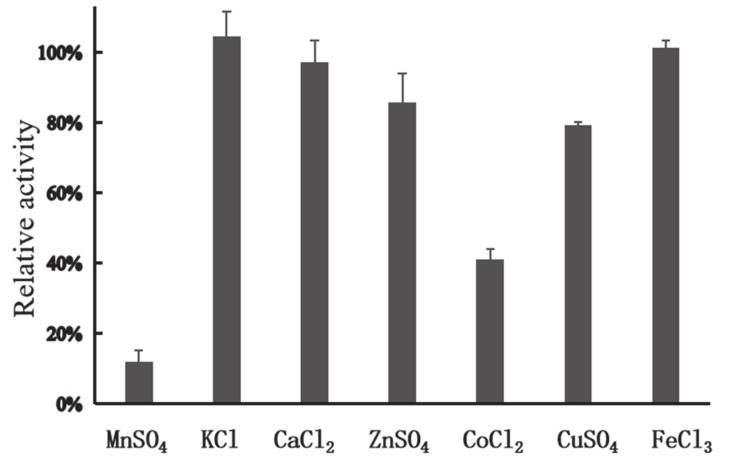
Effect of metal ions on the enzymatic activity of XynT. Effect of different metal ions, including FeCl_3_, MnSO_4_, CaCl_2_, CoCl_2_, KCl, ZnSO_4_, and CuSO_4_ (1 mM), on the activity of XynT was investigated at 40 °C in a MclIvaine buffer (pH 7), using beechwood xylan (1% w/v) as the substrate.

In general, the presence of metals such as Mn^2+^, Cu^2+^, Co^2+^, and Fe^3+^ can have negative impacts on industrial enzymes. Cu^2+^ is involved in the catalysis of auto-oxidation of cysteine residues, resulting in the formation of intermolecular and intramolecular disulfide bonds or the formation of sulfenic acid [29]. Cu^2+^ can also strongly inhibit activity of xylanasesfrom *Sorangium cellulosum* [30], *Geobacillus thermoleovorans* [31], and *Plectosphaerella cucumerina* [32]. Mn^2+^ has negative effects on activity of xylanases from *Lechevalieria* sp. [33] and *Streptomyces viridochromogenes* [34]. Fe^3+^ and Co^2+^ can cause dramatic decrease of activity of xylanases from *G. mesophila* KMM241 [35] and *Sorangium cellulosum* [30]. In this study, among all the metal ions tested, Ca^2+^, K^+^, Zn^2+^, Cu^2+^, and Fe^3+^ (each at 1 mM) showed no apparent effect on the activity of XynT; however, in the presence of Co^2+^, XynT could retain its activity by 40.94% (Figure 3), and, in the presence of Mn^2+^ (1 mM), XynT could retain its activity by 12.08%, indicating that Mn^2+^ had a high negative effect on XynT activity.

**Figure 4:**
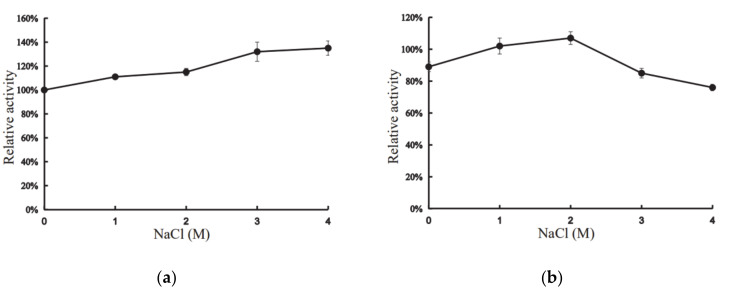
Effect of concentration of NaCl on the activity of XynT. (**a**) XynT activity at different concentrations of NaCl. (**b**) Effect of different concentrations of NaCl on the stability of XynT. The residual activity of the enzyme was measured after incubation in different NaCl concentrations for 24 h.

Interestingly, the presence of 1–4 M NaCl had positive effects on the activity of XynT (Figure 4a). At 4 M NaCl, the activity of XynT increased by 135%, compared to that in the absence of NaCl, and the activity increased with the increase of concentration of NaCl (Figure 4a). XynT was found to be stable in the presence of NaCl: it retained approximately 76% of its activity in 4 M NaCl, and approximately 107% in 2 M NaCl, after 24h incubation at 20 °C (Figure 4b). These characteristics of XynT were similar to those of other cold-active, halotolerant xylanases isolated from various other microbes (Table 1). Thus, XynT belonged to the cold-active, halotolerant xylanase family. Compared with other xylanases, the cold adaption and halotolerancy of XynT are remarkable. Industrial process conditions are often conducted in extreme pHs and temperature, and the presence of salts may inhibit the enzymatic activity [36]. XynT have potential for industrial usage, especially in the processing of sea food and saline food from marine seaweeds.

### 2.3. Kinetic Study of XynT

The hydrolysis of beechwood xylan (10 mg/mL) revealed that with the increase of reaction time, xylanase activity increased in the first 20 min. Therefore, the kinetic parameters were determined at a reaction time of 20 min and at temperatures of 10 °C and 40 °C. The V_max_ of the isolated XynT was 15 μmoL min^−1^ mg^−1^ at 10 °C, and that significantly increased to 62 μmoL min^−1^ mg^−1^ at 40 °C (Table 2). The *K_m_* value for XynT decreased from 22.7 mM to 15.3 mM, as the temperature increased from 10°C to 40 °C. The *k_cat_* values increased with increasing temperature, and the *k_cat_/K_m_* value at 40 °C was 8.3 times higher than that at 10 °C.

### 2.4. Sequence Analysis and Structural Modeling of XynT

The signal peptide analysis revealed that the cleavage site of the signal peptide in XynT lay between Ala21 and Cys22. This cleavage site produced a 358-residue. BLASTp was used to search the mature XynT sequence in the GenBank protein database, and it showed that XynT belongs to the GH10 family, and contains one catalytic domain, and it does not contain a carbohydrate-binding module. The search also showed that XynT and endoxylanase from *Cellvibrio mixtus* share the highest similarity (45.3%).

A previous report has shown that, unlike non-halophilic proteins, halotolerant proteins contain an abundance of acidic amino acids on their surfaces [46]. The high water-binding capacity of these acidic amino acids facilitates the formation of a solvation shell on the protein surface, which can keep them hydrated and assist in the adaptation of the proteins to the high salt concentration-induced environmental pressure [23,47]. A halophilic dihydrolipoamide dehydrogenaseenzyme lost its halo-activity when two of the Glu residues in the interface of the enzyme were replaced by neutral amino acids [48]. It was suggested Glu plays a key role of halo-stablity. XynT contains 26 Asp residues and 35 Glu residues, which accounted for 17.1% of the entire amino acid sequence, and is the highest ratio among all amino acids (Table 3). Xylanase (XynA) from *Zunongwangia profunda* posseses 16.3% acidic amino acids, which activity was significantly increased 140% at 4 M NaCl, however, it only retained ~60% activtiy after 2-h incubation with 4 M NaCl [22]. A halotolerant glucanase from metagenomics of seaweed-associated microbiota posseses 13.8% acidic amino acids, which remained 97% active after a 24-h incubation with 4 M NaCl [11]. These results suggest that high halo-stability of XynT may be associated with its high proportion of acidic amino acids.

**Figure 5:**
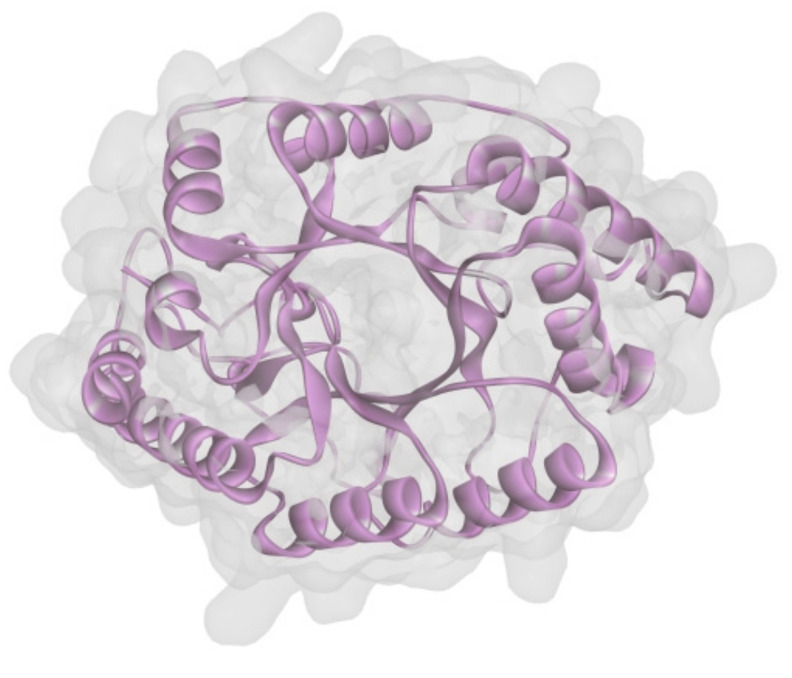
Structure modeling of XynT.

To uncover the structure of XynT, the SWISS-MODEL using the crystal structure of CmXyn10B from *Cellvibrio mixtus* (Protein Data Bank entry: 2cnc.1.A) [49] as the template was employed to build a structural model of mature XynT (residues 22-379). The overall structure of the model had a MolProbity score of 1.73 and a Verify 3D score of 94.8%, indicating that it has good quality. The predicted structure of XynT also possessed the characteristic (β/α)8-barrel fold of GH10 xylanases (Figure 5).

**Figure 6:**
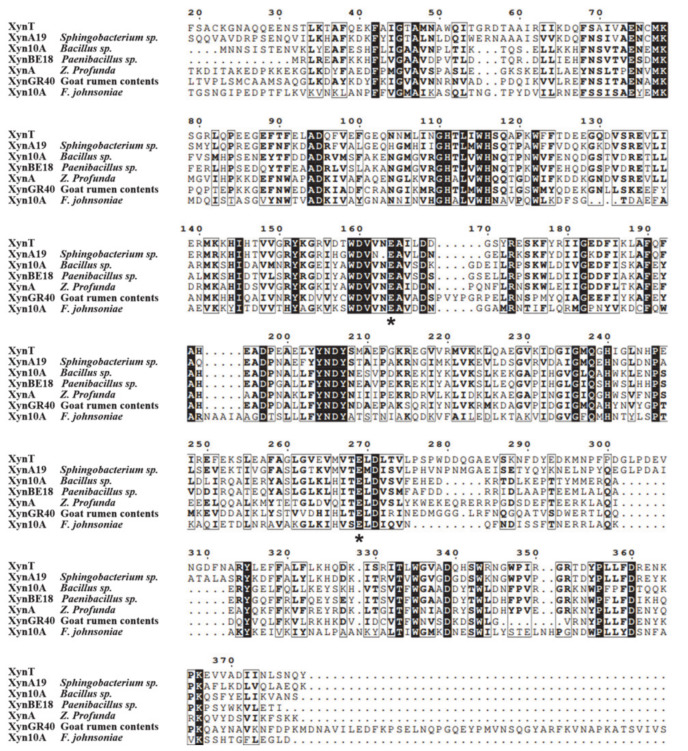
Multiple sequence alignment of XynT with six GH10 cold-active xylanases, including XynA19 from *Sphingobacterium* sp. TN19, Xyn10A from *Bacillus* sp. SN5, XynBE18 from *Paenibacillus* sp. Strain E18, XynA from *Zunongwangia profunda*, XynGR40 from goat rumen, and Xyn10A from *Flavobacterium johnsoniae*. Solid black and grey boxes highlight the identical and similar amino acids, respectively. Two conserved catalytic residues (Glu) are asterisked.

**Figure 7:**
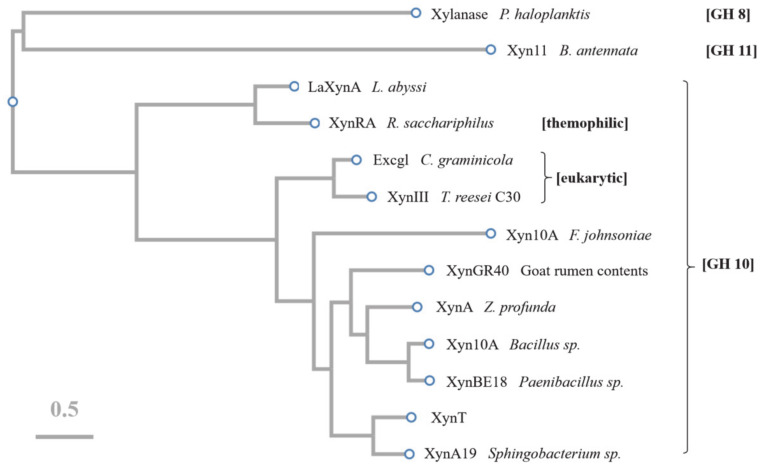
Phylogenetic tree constructed using CLUSTAW of GH11 xylanase (Xyn11) from *Bispora antennata*, GH8 xylanase from *Pseudoalteromonas haloplanktis*, GH10 xylanases (LaXynA) from *Luteinonase abyssi* XH031T, XynRA2 from *Roseithermus sacchariphilus* Strain RA, Excg1 from *Colletotrichum graminicola*, XynIII from *Trichoderma reesei* C30, Xyn10A from *Flavobacterium johnsoniae,* XynGR40 directly from goat rumen, XynA from *Zunongwangia profunda*, Xyn10A from *Bacillus* sp. SN5, XynBE18 from *Paenibacillus* sp. Strain E18, XynA19 from *Sphingobacterium* sp. TN19, and XynT presented in this study.

The amino acid sequence of XynT showed 18.7–56.2% similarity with xylanases from *Sphingobacterium* sp. XynA19 [45], *Bacillus* sp. Xyn10A [39], *Paenibacillus* sp. XynBE18 [44], *Zunongwangia profunda* XynA [22], XynGR40 from goat rumen contents [50], and *Flavobacterium johnsoniae* Xyn10A [41]. Xylanase family 10 enzymes can catalyze the hydrolysis of substrates through the double displacement mechanism, classifying as retaining enzyme. The multiple sequence alignment of six GH10 xylanases, in addition to comparing their crystal structures, showed that the putative catalytic residues of XynT were Glu164 and Glu269 (Figure 6). The amino acid sequences of XynT, one GH11 xylanase, one GH8 xylanase, and ten GH10 xylanases, among which two xylanases are from fungi, one xylanase is thermophilic, and seven xylanases are psychrophilic, were used to construct a phylogenetic tree (Figure 7). The GH8 xylanase and the GH11 xylanase are the far homologs of GH10 xylanases, whereas two other xylanases are the close homologs. Additionally, the thermophilic xylanase and LaXynA are the far homolog of GH10 xylanases, and other seven psychrophilic xylanases are the close homologs. 

XynT also exhibited significant activity even at cold condition. The structural flexibility may contribute to their enhanced enzymatic activity at low temperatures [51]. Some psychrophilic enzymes have low numbers of glycine residues, disulfide bonds, and salt bridges [45]. Similarly, XynT does not contain disulfide bonds, while it contains only 15 salt bridges, and 7.5% Gly residues. Most cold-adapted enzymes, including phosphoglycerate kinase [52], galactosidase [53], and chitobiase [54], have a low Arg/Lys ratio. XynRA2 from *R. sacchariphilus*, which is a thermophilic xylanase with the optimal temperature of 70 °C, has a high Arg/Lys ratio. Several other psychrophilic enzymes have also been reported to have higher Arg/Lys ratios, compared to their thermostable homologs [51]. Similarly to our results, XynT (Arg/Lys ratio = 0.82) and other psychrophilic xylanases had low Arg/Lys ratios, whereas LaXynA from *L. abyssi* had a high Arg/Lys ratio of 6.88 (Table 3). 

## 3. Materials and Methods 

### 3.1. Materials

Table S1 lists the bacterial strains, primers, and plasmids that were used in this study. Plasmids were propagated in *E. coli* DH5α (the transformation hosts) and were subsequently transformed into *E. coli* BL21 (DE3). Substrates including beechwood xylan, birchwood xylan, xylotetraose (X4), xylotriose (X3), and xylobiose (X2) were procured from Megazyme. All other chemicals were of analytical grade.

### 3.2. Growth Conditions, Enzymatic Assay, and Determination of Kinetic Parameters 

*Escherichia coli* was grown in LB medium at 37 °C in a flask shaken at 200 rpm. *Echinicola rosea* JL3085^T^ was grown in 2216E medium (Hopebio, Qingdao, China) at 30 °C in a flask shaken at 200 rpm [55]. Determination of xylanase activity has been previously described [39]. The kinetic parameters, *K_m_*, V_max_, and *k_cat_*, were determined according to a previously described method [37].

### 3.3. Cloning, Expression and Purification of E. roseaxynt in E. coli

General molecular biology techniques have been described previously [56]. The pET-22b(+) plasmid was linearized using EcoRI restriction enzyme (TaKaRa, Ohtsu, Japan). The gene sequence of *xynT* was amplified from genomic DNA using the primers XynT_F and XynT_R. The xynT gene was inserted into the linearized pET-22b(+) using Seamless cloning kit (Beyotime, Shanghai, China) to construct the expression plasmid (pET22b_xynT), and the heat shock method was then used to transform the plasmid into *E. coli* BL21 (DE3). The restriction digestion, along with DNA sequencing using an ABI3100 (Applied Biosystems, Foster City, California), were used to identify the transformants. Next, the positive transformant (transformant containing pET22b_xynT) was grown in LB medium at 37 °C in the presence of 100 μg/mL ampicillin (Merck, Germany), until an OD_600_ of 0.6 was achieved. Isopropyl-β-d-1-thiogalactopyranoside (IPTG, 1 mM) was added at 25 °C for 12 h, to induce protein expression.

The sample was centrifuged at 12000× *g* and 4 °C for 5 min, the cultured supernatant was collected and concentrated using a Pierce^TM^ protein concentrator (PES 5K MWCO; ThermoFisher). The recombinant protein was purified from the concentrated supernatant through its His-tagged sequence using the BeyoGold^TM^ his-tag purification resin (Beyotime, Shanghai, China). The recombinant XynT was separated by SDS–PAGE to determine its molecular mass and purity. The Bradford assay kit was used to determine the protein concentration (Beyotime, Shanghai, China).

### 3.4. Effect of pH on the Stability of Recombinant XynT

The optimal pH for the enzymatic activity of XynT was determined using buffers with pH 4–10 at 40 °C, and beechwood xylan (1% w/v) was used as the substrate. The enzyme was incubated in buffers with different pH at 4 °C for 2 h and its pH stability was determined based on its residual activity at pH 7. The buffers that were used for this enzymatic assay were McIlvaine buffer, pH 4–8 and 50 mM glycine-NaOH buffer, pH 9–10.

### 3.5. Effect of Temperature on the Stability of Recombinant XynT

Thermostability was determined in MclIvaine buffer (pH 7) using beechwood xylan (1% w/v) as the substrate at a temperature range of 0 to 70 °C. The residual activity of the enzyme was measured at 40 °C post-incubation for 2 h at 0–60 °C.

### 3.6. Effect of Metal Ions on the Activity of Recombinant XynT

The effects of different metal ions, including FeCl_3_, MnSO_4_, CaCl_2_, CoCl_2_, KCl, ZnSO_4_, and CuSO_4_ (each at a final concentration of 1 mM), on the activity of XynT were investigated at 40 °C in McIlvaine buffer (pH 7), using beechwood xylan (1% w/v) as the substrate.

### 3.7. Effect of Salt on the Activity of Recombinant XynT

The effects of different salt concentrations, 0–4 M NaCl, on xylanase activity was evaluated at pH 7 and 40 °C. The halotolerance of the enzyme was evaluated by incubating the enzyme at 20 °C in the presence of 0–4 M NaCl for 24 h, and then measuring its residual activity at 40 °C.

In analysis of hydrolysis, the purified XynT was incubated with beechwood xylan or birchwood xylan at 40 °C in McIlvaine buffer (pH 7) for 2 h or 8 h. Thereafter, the enzyme was removed using a Pierce^TM^ protein concentrator (PES 5K MWCO; ThermoFisher). A high-performance anion-exchange chromatography (HPAEC, ICS-3000 system) equipped with a CarboPac MA1 column (Dionex, CA, USA) was used to analyzed the enzyme, and 0.1 M NaOH and 0.2 M NaAc were used as the solvents.

### 3.8. Putative Structure Analysis

The BLASTp program was used to search the amino acid sequence of XynT against the NCBI database. Homology modeling using SWISS-MODEL was used to generate a three-dimensional model of XynT [57]. The assessment of the predicted model was carried out on Molprobity and Verify 3D [58,59]. SignalP 5.0 was used for signal peptide prediction [60]. Multiple sequence alignment was performed using CLUSTALW and ESPript3.0 [61]. Disulfide bonds and salt bridges (distances ≤ 3.2 Å), were predicted based on a previously described method [12,62]. The model structure was analyzed by Discovery studio software. 

## 4. Conclusions

In this study, we cloned xynT, a novel xylanase gene from a halotolerant bacterium *Echinicola rosea* sp. nov. JL3085^T^. Biochemical characterization and structural analysis showed that XynT protein belonged to the cold-active, halotolerant xylanase family, and exhibited a high catalytic activity at high salt concentrations (4 M NaCl), among other known GH family 10 psychrophilic xylanases. XynT could digest xylan to produce smaller oligosaccharides, without producing xylose, thus is suitable for the production of XOS, which could improve the growth prebiotic bacteria of *Lactobacillus* and *Bifidobacterium*. XOS of various polymerization degrees would have more potential functions, which is governed by their substitution patterns [23]. But it has been difficult to produce specific XOS using some chemical and physical methods. Enzymatic transformation of xylan into XOS using XynT is a promising technical route for production of various substituted XOS. The presented XynT may be used to study the structure-function relationship in cold-active enzymes, and have potential applications in the bleaching of paper and pulp, bioremediation, food, and the human health sector, especially in seaweeds and medicine processing as a tool to prepare prebiotics.

## Figures and Tables

**Table 1:** Biochemical characteristics of xylanases.

Xylanase (Source/Microorganism)	GH	T_opt_ °C	pH_opt_	Activity in the Presence of 4 M NaCl	Residual Activity at Low Temperature	Refs
XynT (*Echinicola rosea* sp. nov. JL3085^T^)	10	40	7	135%	26%, 0 °C; 51%, 5 °C	This study
XynA (*Zunongwangia profunda*)	10	30	6.5	140%	23%, 0 °C; 38%, 5 °C	[22]
LaXynA (*Luteinonas abyssi* XH031^T^)	10	40	7	100%	51%, 10 °C	[37]
XynRA2 (*Roseithermus sacchariphilus* Strain RA)	10	70	8.5	94%	<30%, 20 °C	[38]
Xyn10A (*Bacillus* sp. SN5)	10	40	7	<50%	~30%, 5 °C; ~30%, 10 °C	[39]
Excg1 (*Colletotrichum graminicola*)	10	65	5.5	~100% in 3 M NaCl		[40]
Xyn10A (*Flavobacterium johnsoniae*)	10	30	8		50%, 4 °C	[41]
Xyn11 (*Bispora antennata*)	11	35	5.5		20%, 0 °C; ~40%, 10 °C	[42]
XynGR40 (goat rumen contents)	10	30	6.5		10%, 0 °C	[12]
Xylanase (*Pseudoalteromonas haloplanktis*)	8	35	5.3–8		60%, 5 °C	[43]
XynBE18 (*Paenibacillus* sp. Strain E18)	10	50	7–9		~30%, 30 °C	[44]
XynA19 (*Sphingobacterium* sp. TN19)	10	45	6–6.5		<10%, 0 °C	[45]

T_opt_: optimal temperature; pH_opt_: optimal pH; GH: glycoside hydrolase family.

**Table 2:** Kinetic parameters of XynT in hydrolysis of beechwood xylan.

Temperature °C	V_max_ μmoL min^−1^ mg^−1^	*K_m_* mM	*k_cat_* S^−1^	*k_cat_*/*K_m_* S^−1^ mM
10	15 ± 0.7	22.7 ± 2.7	9 ± 0.4	0.4
40	62 ± 1.5	15.3 ± 1.3	51 ± 1.3	3.3

**Table 3:** Amino acid composition and putative parameters affecting the stability and flexibility of xylanases.

Composition/Parameter	XynT	XynA	XynGR40	XynA19	Xyn10A	xylanase	XynRA2	LaXynA
Percent Gly (%)	7.5	5.3	7.6	6.8	5.2	7.3	9.3	9.9
T_opt_ (°C)	40	30	30	45	40	35	70	40
Arg/Lys ratio	0.82	0.38	0.5	0.52	0.25	0.43	5.3	6.88
Percent alkaline amino acid (%)	13.6	17.9	13.5	15.2	11.4	10.6	7.4	9.3
Percent acidic amino acid (%)	17.1	16.3	13.7	13	11.4	8	14.7	14.2
Reference	This study	[22]	[12]	[45]	[41]	[43]	[38]	[37]

T_opt_: optimal temperature. Alkaline amino acids include Arginine (Arg), Histidine (His) and Lysine (Lys). Acidic amino acids include Aspartic acid (Asp) and Glutamic acid (Glu).

## Supplementary Materials

The following are available online at https://www.mdpi.com/1660-3397/18/5/245/s1, Table S1: Bacterial strains, plasmids, and primers used.
