# Supplementary material for: Isolation and Characterization of a Novel Cold-Active, Halotolerant Endoxylanase from Echinicola rosea Sp. Nov. JL3085T
Source: Mar Drugs. 2020 May 6;18(5):245. doi: 10.3390/md18050245 (PMC7281462; doi:10.3390/md18050245)
Supplement: Supplementary file 1 [file marinedrugs-18-00245-s001.pdf]

Table S1. Bacterial strains, plasmids, and primers used.

Strain/plasmid/primer	Characteristic(s)	Reference
Bacterial strains		
E. coli DH5α	F$^-$ *φ80dlacZΔM15 Δ(lacZYA-argF)U169 deoR recA1 endA1 hsdR17(rK$^-$, mK$^+$) phoA supE44 λ$^-$ thi-1 gyrA96 relA1*	Takara
E. coli BL21 (DE3)	F$^-$ *ompT hsdSB(rB- mB-) gal dcm(DE3)*	Sigma
Echinicola rosea JL3085^T	Wild-type halotolerant bacterium	[54]
Plasmids[a]		
pET-22b(+)	Amp^{r} [a]; T7lac promoter	Invitrogen
pET22b_xynT	pET-22b(+); Amp^{r}; carries *E. rosea xynT*	This study
Primers [b]		
XynT_F	*tcggaattaattcggatccg*ATGGTGGGAGCTTATCAAAC	This study
XynT_R	*aagcttgtcgacggagctcg*gtggtggtggtggtggtgCTAGTATTGATTTGATAAAT	

[a] Amp^{r}: resistance to ampicillin.
[b] Italic sequences indicate the complementary sequences to the EcoRI restriction sites on pET-22b(+) plasmid.
[c] Underline sequences indicate the nucleotides encoding the His-tag sequence.
